# Supplementary material for: Multiplex enCas12a screens detect functional buffering among paralogs otherwise masked in monogenic Cas9 knockout screens
Source: Genome Biol. 2020 Oct 15;21:262. doi: 10.1186/s13059-020-02173-2 (PMC7558751; doi:10.1186/s13059-020-02173-2)
Supplement: Supplementary file 1 — Additional file 1: Fig. S1. Comparison of computationally derived hits from our analysis with hits in De Kegel and Ryan et al. [15]. Fig. S2. (A) Knockout of GPF with a crRNA targeting GFP in enCas12a knock-in cells. (B) Targeting two cell surface markers with a dual-guide crRNA in enCas12a-expressing OVCAR8 cells. (C) Total amplicon reads for the paralog screen. Dashed line indicates 500x sequencing depth (6 m reads for 12 k library). (D) Distribution of reads (boxplot) indicates good library representation in each sample. Dashed line = 30 reads/construct. (E) Clustering of normalized read counts. Clustering of replicates is consistent with high-quality screen data. (F, G) Lack of positional bias in mirror constructs containing the same two crRNA in A-B and B-A orientations. (H, I) SMF in this screen vs. BF from Avana data.(J) Z-transformation of distribution of dLFC (zdLFC) after truncating top/bottom 2.5% of values approximates a normal distribution. Fig. S3. Comparison of zdLFC scores to scores generated by GEMINI. (A) zdLFC vs GEMINI scores for 24 synthetic lethal pairs with their respective correlation coefficients. (B) zdLFC vs GEMINI scores for all tested paralog pairs with their respective correlation coefficients. Fig. S4. Comparison of common paralog pairs tested in our enCas12a screen with the CHyMErA screens. (A) Comparison of the 12 enCas12a hits in this study that were screened in HAP1 in the CHymErA study. (B) Comparison of all 110 paralog pairs tested in both enCas12a screen and the HAP1 CHymErA screen. Fig. S5. Gaussian mixture modeling (GMM) of gene expression of Avana 19Q4 cell lines. (A) Scatter plot of standard deviation of expression versus mean expression of gene assayed in Avana library in Avana19Q4 cell lines. (B) Contour plots of the two Gaussians from a two-component mixture model of data shown in A. (C) Contour plots of three-component GMM. (D) Contour plots of fourcomponent GMM. [file 13059_2020_2173_MOESM1_ESM.pdf]

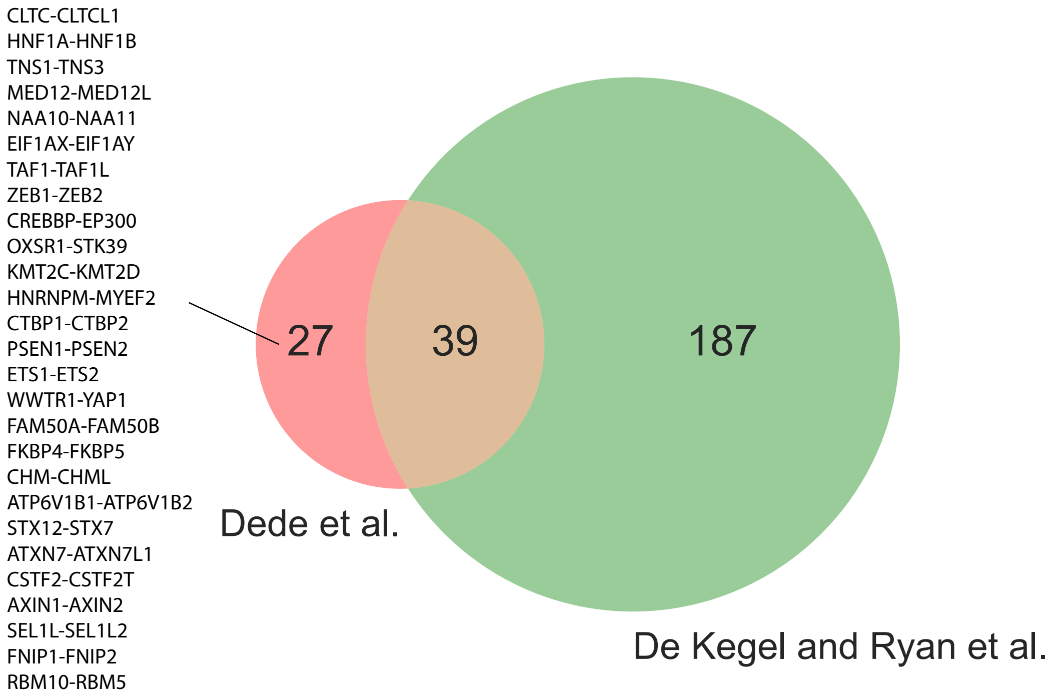

Fig. S1. Comparison of computationally derived hits from our analysis with hits in De Kegel and Ryan et al (15).

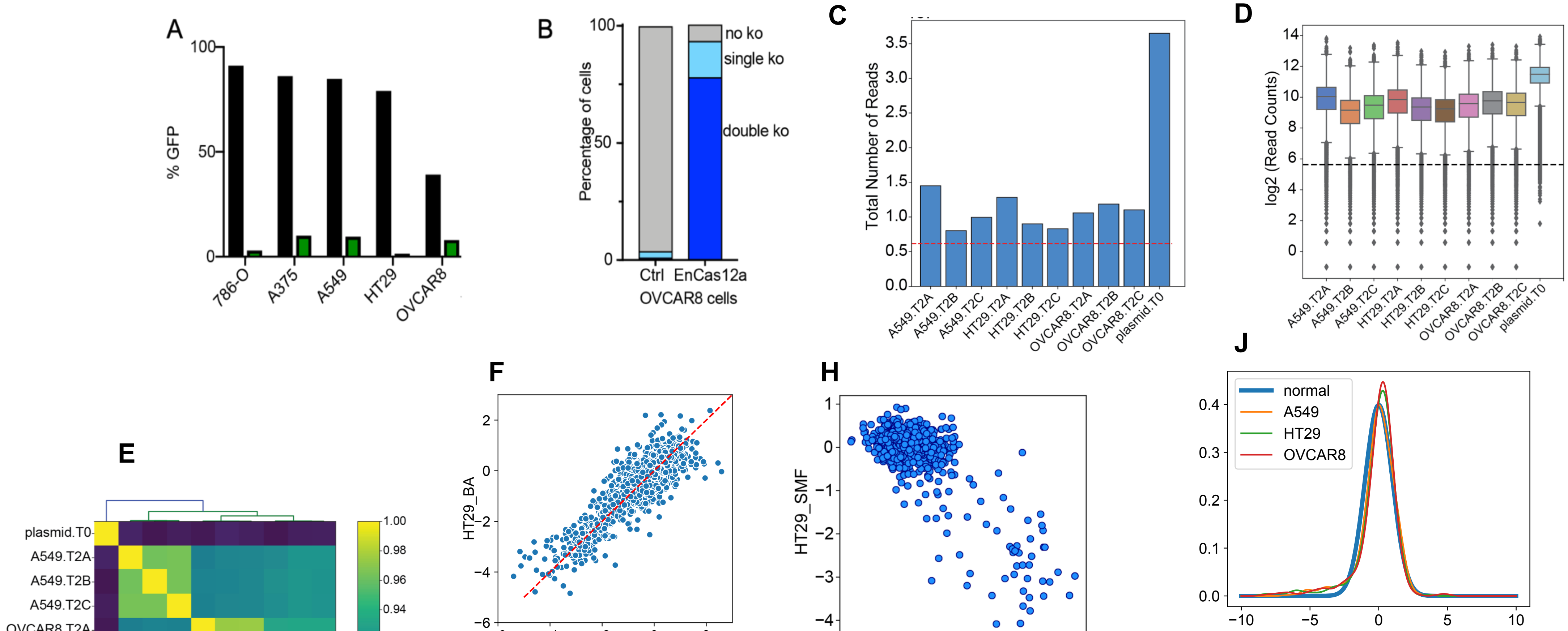

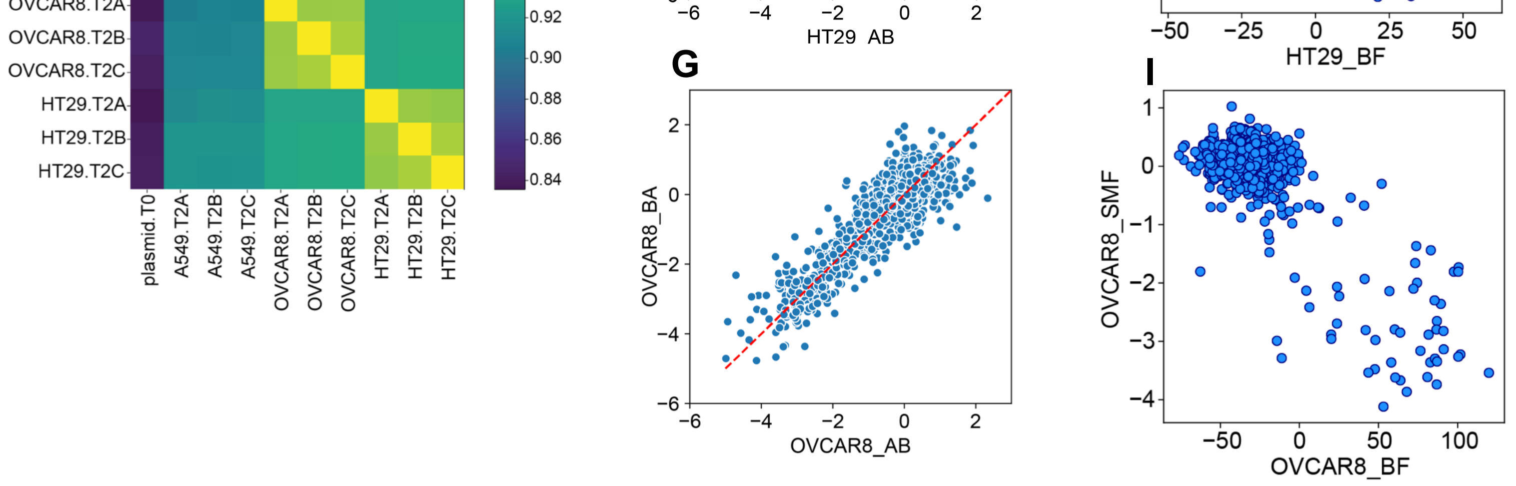

Fig S2. (A) Knockout of GPF with a crRNA targeting GFP in enCas12a knock-in cells. **(B)** Targeting two cell surface markers with a dual-guide crRNA in enCas12a-expressing OVCAR8 cells. **(C)** Total amplicon reads for the paralog screen. Dashed line indicates 500x sequencing depth (6m reads for 12k library). **(D)** Distribution of reads (boxplot) indicates good library representation in each sample. Dashed line = 30 reads/construct. **(E)** Clustering of normalized read counts. Clustering of replicates is consistent with high-quality screen data. **(F, G)** Lack of positional bias in mirror constructs containing the same two crRNA in A-B and B-A orientations. **(H, I)** SMF in this screen vs. BF from Avana data.**(J)** Z-transformation of distribution of dLFC (zdLFC) after truncating top/bottom 2.5% of values approximates a normal distribution.

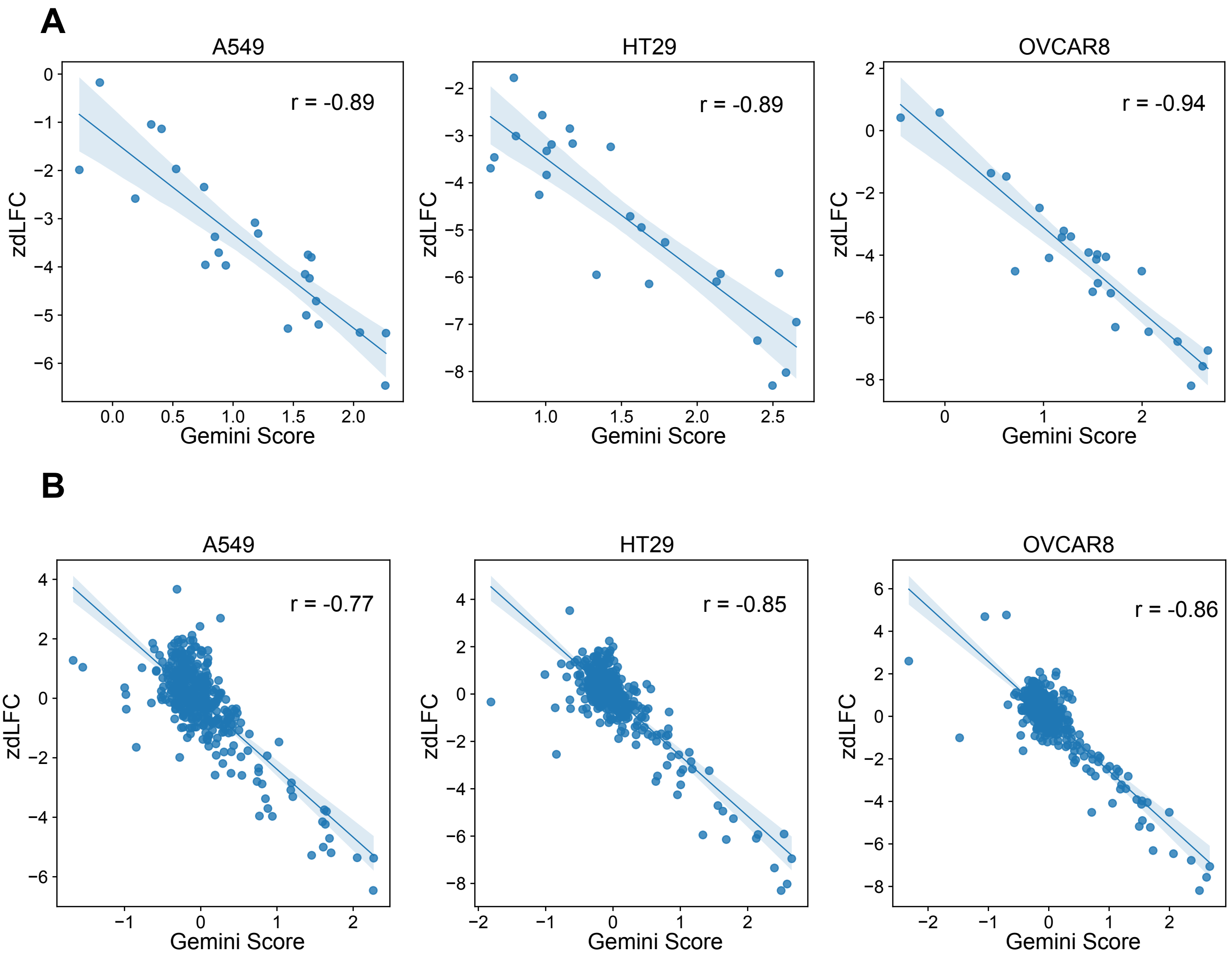

Fig S3. Comparison of zdLFC scores to scores generated by GEMINI. (A) zdLFC vs GEMINI scores for 24 synthetic lethal pairs with their respective correlation coefficients. (B) zdLFC vs GEMINI scores for all tested paralog pairs with their respective correlation coefficients.

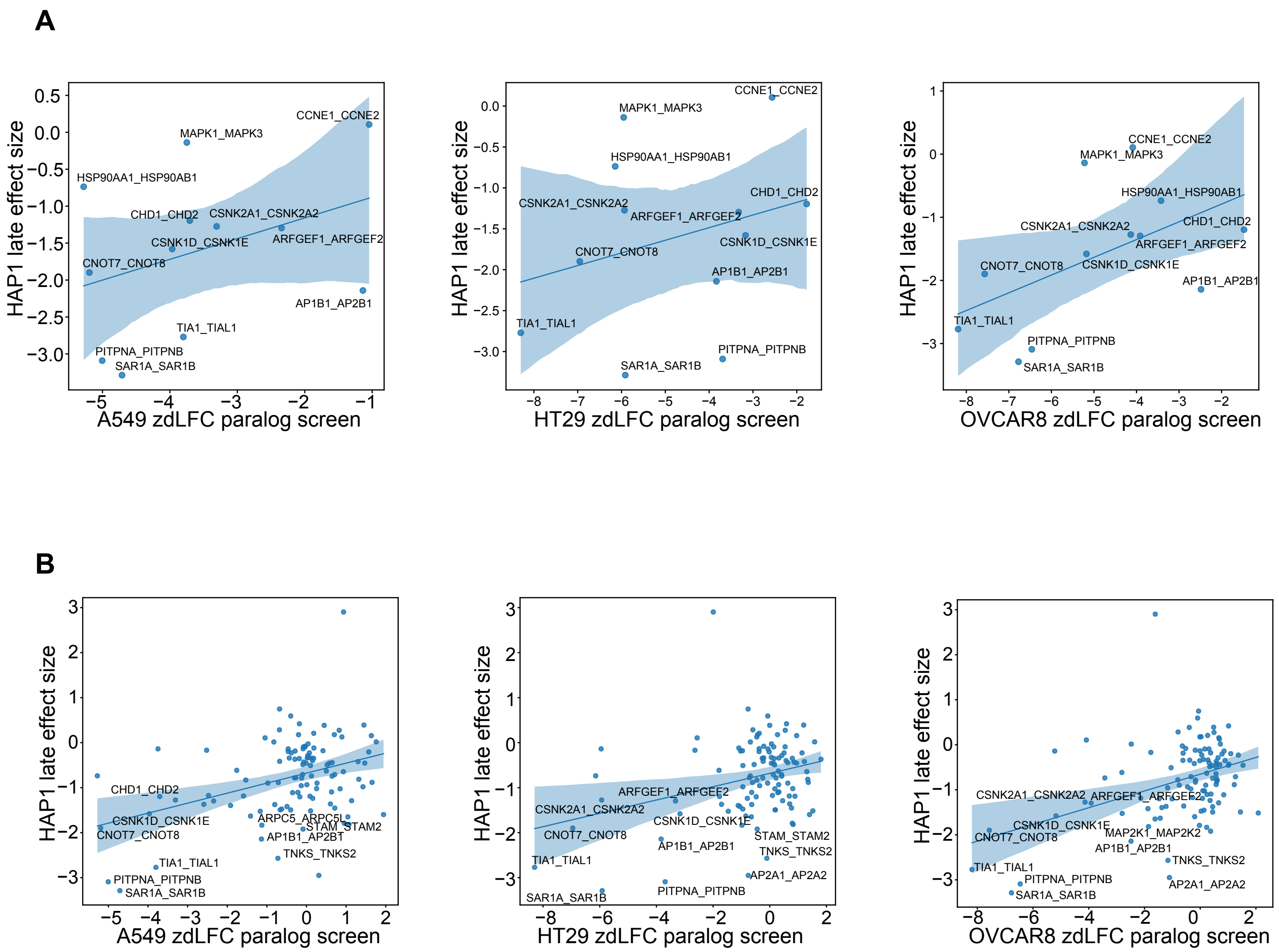

Fig. S4. Comparison of common paralog pairs tested in our enCas12a screen with the CHyMErA screens. (A) Comparison of the 12 enCas12a hits in this study that were screened in HAP1 in the CHymErA study. **(B)** Comparison of all 110 paralog pairs tested in both enCas12a screen and the HAP1 CHymErA screen.

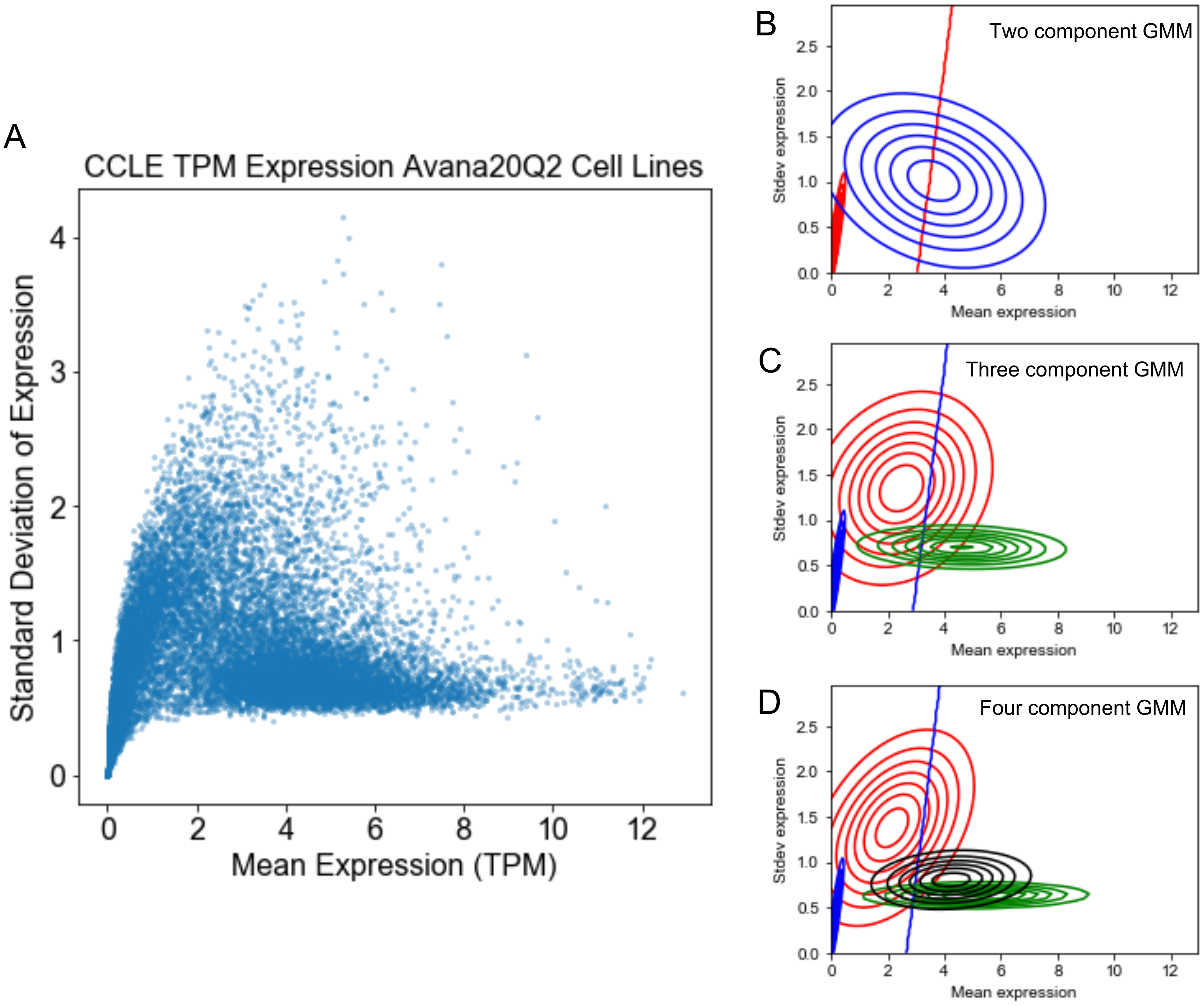

Fig S5. Gaussian mixture modeling (GMM) of gene expression of Avana 19Q4 cell lines. **(A)** Scatter plot of standard deviation of expression versus mean expression of gene assayed in Avana library in Avana19Q4 cell lines. **(B)** Contour plots of the two Gaussians from a two-component mixture model of data shown in A. **(C)** Contour plots of three-component GMM. **(D)** Contour plots of four-component GMM.
