# Supplementary material for: Multiplex enCas12a screens detect functional buffering among paralogs otherwise masked in monogenic Cas9 knockout screens
Source: Genome Biol. 2020 Oct 15;21:262. doi: 10.1186/s13059-020-02173-2 (PMC7558751; doi:10.1186/s13059-020-02173-2)
Supplement: Supplementary file 6 — Additional file 6. Review history. [file 13059_2020_2173_MOESM6_ESM.docx]

Review History

**First round of review**

**Reviewer 1**

**Are you able to assess all statistics in the manuscript, including the appropriateness of statistical tests used?** Yes, and I have assessed the statistics in my report.

**Comments to author:**

Notes on peer review for GBIO-D-20-00707 (M. Dede, M. McLaughlin, E. Kim, T. Hart) for Genome Biology.

The manuscript starts by reviewing evidence from the S. cerevisiae literature and observing that in yeast, genes without paralogs are more than twice as likely to be judged essential when compared to genes with paralogs (50-51).  Inspired by this observation, they use publicly available genome wide screen data from the Cancer Dependency Map to investigate paralogs among constitutively expressed genes classified as "never-essential" to determine which if any of these paralog pairs are synthetic lethal when simultaneously knocked down.  After identifying a candidate list of ~400 such paralog pairs, they perform a CRISPR/enCas12a multiplex dual-knockout screen in three cancer cell lines (A549, HT29, OvCar8) to identify synthetically lethal pairs among the paralogs targeted.

Overall, this is a worthwhile investigation and addresses an important question in functional genomics. It shows a promising application of Cas12a technology as a platform for dual guide screens. However, there are a number of questions to be addressed in order to clarify the methods applied and put the work into context within the current literature.  

General comments:

1.  How many of the selected paralog pairs are true ohnologs, that is are paralog pairs with evidence of a whole-genome duplication (WGD) event?

2.  The authors mention (page 4, line 92) that "Interestingly, among constitutively expressed genes, many are never essential in any CRISPR knockout fitness screen". How is essentiality defined here? Is it simply using a threshold on the CERES score, or using the authors' proposed Bayes Factor (BF) score? If so, what threshold was used?  This should be specified throughout the text in brackets when appropriate.

3.  The sgRNA selection (which on- and off-target rules were used to select best guides for a given pair of genes, for instance) is nowhere discussed in the manuscript. This should be included and discussed at length since the quality of the results might be impacted by the quality of sgRNA design. I would imagine that for 2 genes that are highly homologous, say with a sequence similarity at the protein level greater than 95% it may be hard to design guides that uniquely target one paralog or the other, therefore impacting the ability of measuring single knockout effects. Is that a problem that the authors encountered?

4.  For estimating single KO effects, the authors include constructs in the library in which they pair the target gene guides with guides targeting known nonessential genes (line 155).
-- How were the non-essential genes selected?
-- How were the guides targeting the non-essential genes selected?
-- How many (target x neg) constructs were designed for each target gene?
-- Was the same set of non-essential guides used across target genes?

5. A description of the methodology to go from sequencing reads to a matrix of counts is missing. Among others, the following information is missing:
-- What software was used to process sequencing reads? What were the parameters? What was the quality of the sequencing?
-- Which software (or programming language) was used to analyze the count data?
-- Was any normalization performed?
-- Were constructs with low counts in the plasmid library filtered out?

6. In Figure 3F, the bulk of the distributions of the dLFC is shifted below 0 for all three cell lines. What is the reason behind this shift?

7. How does the current work contrast to previously published CRISPR screens targeting pairs of paralogs? Previous work should be cited and discussed, for instance https://doi.org/10.1038/s41587-020-0437-z.  The results of the present manuscript should be compared to he CRISPR-based screens from https://doi.org/10.1038/s41587-020-0437-z, at least for the pairs of genes that are common to both screens, and also contrasted to the dropout screen described in https://www.nature.com/articles/nbt.3834, which uses a comprehensive paired-gene library.

8. The difference between the average dKO effects and the sum of sKO effects (dLFC) has been shown to be a suboptimal measure of synergy (https://doi.org/10.1186/s13059-019-1745-9) since it does not take into account guide-level variability. I strongly recommend the authors to use a statistical model that takes into account guide-level variability in the estimation of synergy scores, such as the GEMINI tool (https://doi.org/10.1186/s13059-019-1745-9), or using the methodology described in https://www.nature.com/articles/nbt.3834.

9. The Gaussian Mixture Modeling (Methods; 322-333) appears odd in that looking at Figure 1a, the "Sometimes" group (blue) does not look at all bivariate Gaussian, while the "Constitutive" group (orange) looks as if half its ellipsoid is empty.  If this is used only to filter the list of paralog pairs, this may be relatively harmless (causing some false negatives in the discovery process, but not likely false positives) -- basically drawing a rectangle out to the right along the mean expression axis and low on the SD axis.  Can the authors please comment on the intended purpose of the GMM within this work?

10. Robust Z-score (~179-182): Details on precisely what this means?  Is there normalization by a standard error, and if so, which one?  I didn't see details in the Methods section.  Was this based on winsorizing the dLFC values and centering and scaling them, or something else?  We did not see these details.

11. Line 232: Is the factor "3" from comparing "27.9%" to "11.6%"?  If so, suggest "2.4" instead of "3".  If not, where does "3" come from?

12. Methods (341-346): "... the percent sequence identities of all human target genes to their paralogs were plotted against the percent sequence identities of the paralogs to the target human gene to reveal that the majority of the human paralogous gene pairs had low percentage sequence similarity."  It sounds as if they are getting the percent similarity of "B" to "A", then of "A" to "B", then plotting them against each other?  If so, this sounds good, but this description is a little confusing -- consider rewording?

13. Possible typos or awkward constructions:
-- Line 150: "We manually five additional candidate genes…"
-- LIne 184: "Show characteristics lethal synthality…"
-- Line 345: "... paralog pairs which were both in always expressed gene list"
-- Line 359-360: "...synthetic essentiality which is defined that a gene is essential when the other paralog partner lost the function…."

**Reviewer 2**

**Are you able to assess all statistics in the manuscript, including the appropriateness of statistical tests used?** Yes, and I have assessed the statistics in my report.

**Comments to author:**

See joint review with Reviewer 1.

**Reviewer 3**

**Are you able to assess all statistics in the manuscript, including the appropriateness of statistical tests used?** Yes, and I have assessed the statistics in my report.

**Comments to author:**

In this manuscript the authors explore the role of functional buffering provided by the paralogs of a gene in compensating its activity when that gene is inactivated via CRISPR-cas9 targeting. Thus, shedding light on how this prevents constitutively expressed genes to be detected as 'essential' for cellular survival in large scale mono-genic knock down screens.

The authors build on the assumption that constitutively expressed genes are essential in at least some context and that combinations of environmental or genetic buffering masks the impact of their experimental inactivation on cellular fitness. By mining public available cancer dependency datasets, encompassing gene essentiality profiles of hundreds of cell lines, the authors found that paralog gene pairs are over represented among constitutively expressed genes that are never called as significantly essential, i.e. not impacting cellular fitness upon CRISPR-cas9 targeting. This supports the authors hypothesis hinting that the genetic buffering compensating the inactivation of constitutively expressed genes is provided by their paralogs.

The authors then reasoned that simultaneously probing cellular models for their dependency on paralog pairs might offer an opportunity to identify new synthetic lethal gene pairs that might be exploited for the development of new cancer therapies.

To this aim, the authors have selected 400 hundreds candidate paralog gene pairs, according to different criteria and performed a dual knock out screen targeting each pair in 3 immortalised human cancer cell lines through a enCas12a dual knockout system. 24 of the tested pairs resulted into a synthetic lethality in at least 2 of the tested models and are described in dept by the authors.

Briefly, this manuscript describes important findings that might have an impact on how outcomes from monogenic knock out screens will be interpreted and illustrates a strategy for identifying promising synthetic lethality to be further confirmed via dedicated dual knock out screens. Genetic interaction screens are expected to be increasingly performed in the future in human cells, so providing guidance on which are the gene pairs to be prioritised for testing across a so large spectrum it is timely and important.

However, the hypothesis tested by the authors has been already investigated, using a similar approach, in [PMID: 31652272]. In addition the selection of 400 gene pairs tested with the dual knock out screen performed by the authors is not guided by the CRISPR based landmark analysis nor the buffering association study. As a consequence, it seems to me that the analyses reported in the first part of the results and figures 1 and 2 are just confirmatory and could be totally removed.

In addition, the title states that Multiplex enCas12a screens show… etc etc. whereas this is demonstrated by the authors via mining existing data. As a consequence the title should be changed.

The results from the dual knock out screen remains potentially very useful and potentially impactful but further functional characterisation of the identified synthetic lethal gene pairs should have been performed.

In conclusion, I do not believe that this manuscript would be suitable for publication in Genome Biology at this stage. The author should clarify scope and aim of this manuscript. I would definitely reshape this submission and focus it on the dual screen effort, whose outcomes deserve definitely a closer look.

In addition, I have the following major points:
* From the main text it seems that the author detected unbiasedly three major populations of genes based on their mean basal expression (and corresponding variability) across cell lines. By looking at figure 1A and reading the methods, it is clear that the number of Gaussian distributions to be used was decided a priori by the authors. This should be made clear in the main text or a numerical justification for this choice should be provided.  In addition, from figure 1A it seems that several points are drawn from the 'orange distribution' even if they have a mean expression level proximal to 0 and a quite low standard deviation. Can these genes be really considered constitutively expressed?

* The author call the 'sometimes expressed genes' tissue-specific. I don't think that this is justified in any way. Some of these genes could be just expressed in minority of cell lines across different tissues, or ubiquitous subtypes with a common mutated oncogene, thus same set of active transcription factors

* no statistical estimation is provided with respect to paralog enrichments across the different classes of genes in figure 1B, other than percentages. This should be included.

* FDRs and effect size indicators for the tested buffering interactions reported in figure 2 should be provided

* The author should discuss the possibility of the identified synthetic lethal gene pairs being context-specific, and how the genomic heterogeneity of the cell line selected for their study might have impacted the results. Furthermore, which criteria were used for selecting those three cell lines?

* The Broad DepMap dataset is slightly outdated. A 20Q2 version is now available. The authors might consider revamping their analysis with this more recent version or (even better) by using a recent joint dataset of cancer dependencies integrating both Sanger and Broad CRISPR screens (available at https://depmap.org/broad-sanger/

* I have definitely a problem with the title of this manuscript as it also suggests to me the opposite of the finding presented by the authors.  Doesn't 'functional buffering systematically absent in genome-wide CRISPR/Cas9 screens' imply that the effect of the buffering is absent therefore in such screens paralogs can be detected as essential genes? What about to replace this with something more like: "Functional buffering prevents detecting fitness genes among paralogs in genome-wide CRISPR/Cas9 etc. etc."?

Minor points:
* The color scheme in figure 1B could be improved, for example I cannot decide if the top bar in the 'sometimes expressed genes' column corresponds to common or context essential.

**Authors Response**

Dear Editors,

We would like to thank the reviewers for their careful and critical analysis of the manuscript. To further solidify the findings proposed in the paper, we have made the following modifications in our manuscript as outlined in the point-by-point responses to the reviews. We believe that these modifications address the reviewers’ concerns and have improved our manuscript.

***Reviewer #1:***

*The manuscript starts by reviewing evidence from the S. cerevisiae literature and observing that in yeast, genes without paralogs are more than twice as likely to be judged essential when compared to genes with paralogs (50-51). Inspired by this observation, they use publicly available genome wide screen data from the Cancer Dependency Map to investigate paralogs among constitutively expressed genes classified as "never-essential" to determine which if any of these paralog pairs are synthetic lethal when simultaneously knocked down. After identifying a candidate list of ~400 such paralog pairs, they perform a CRISPR/enCas12a multiplex dual-knockout screen in three cancer cell lines (A549, HT29, OvCar8) to identify synthetically lethal pairs among the paralogs targeted.*

*Overall, this is a worthwhile investigation and addresses an important question in functional genomics. It shows a promising application of Cas12a technology as a platform for dual guide screens. However, there are a number of questions to be addressed in order to clarify the methods applied and put the work into context within the current literature.*

We appreciate the reviewer’s positive comments in support of our manuscript and have addressed the detailed concerns below. We believe that these changes have improved the manuscript.

*General comments:*

*1. How many of the selected paralog pairs are true ohnologs, that is are paralog pairs with evidence of a whole-genome duplication (WGD) event?*

We thank the reviewer for their question. To address this issue, we utilized the OHNOLOGS v2.0 database, which is a repository of genes arising from whole genome duplications (Singh et al., 2015). We obtained the ohnolog pairs for human genes using the “Strict” threshold described in the database with the criterion of q-score (outgroups) < 0.001 AND q-score (self comparison) < 0.001. We observed that 246 out of 405 paralog pairs we tested are ohnologs (60.7%) according to this strict threshold. For the intermediate threshold in the same database (q-score(outgroups) < 0.01 AND q-score(self comparison) < 0.01), 279 out of 405 paralog pairs (68.9%) were found to be ohnologs. Out of the 24 synthetic lethal paralog pairs we identified, 15 (62.5%) are ohnologs according to the strict threshold, while 16 pairs (66.7%) are defined as ohnologs according to the intermediate threshold. We have added additional description to the text.

*2. The authors mention (page 4, line 92) that "Interestingly, among constitutively expressed genes, many are never essential in any CRISPR knockout fitness screen". How is essentiality defined here? Is it simply using a threshold on the CERES score, or using the authors' proposed Bayes Factor (BF) score? If so, what threshold was used? This should be specified throughout the text in brackets when appropriate.*

We appreciate the reviewer pointing out this oversight on our part. Here, essential genes are defined as genes with a Bayes Factor (BF) of greater than or equal to 10, which corresponds to a probability of essentiality of 99%. We have added additional description to the text.

*3. The sgRNA selection (which on- and off-target rules were used to select best guides for a given pair of genes, for instance) is nowhere discussed in the manuscript. This should be included and discussed at length since the quality of the results might be impacted by the quality of sgRNA design. I would imagine that for 2 genes that are highly homologous, say with a sequence similarity at the protein level greater than 95% it may be hard to design guides that uniquely target one paralog or the other, therefore impacting the ability of measuring single knockout effects. Is that a problem that the authors encountered?*

We used a library design from John Doench’s lab (De Weird et al) and we have added an extensive description in the methods section of our library design, sequencing, and sequence analysis approaches. Moreover, all of our code and data are available on Figshare

The new methods section is as follows:

***Library design***

**We selected Cas12a crispr RNA sequences from a library from [DeWeirdt et al]. Guides were selected from an AsCas12a library design from July 2019, representing an intermediate phase of development of the DeWeirdt et al work. Up to the top three guide sequences were selected from the library for each of the 793 candidate paralog genes (405 gene pairs), but given the restrictive TTTV PAM sequence for AsCas12a, three guides were not available for every gene. For two genes (ABHD16B, DGCR6), no crRNA were present in the library; pairs including these genes were removed in the downstream analysis. As controls, a set of 50 nonessential and 50 pan-essential genes were chosen; genes were filtered for those with 1:1 orthologs in both rat and mouse to provide a useful multi-species reference set. These control genes are listed in Supp Table 4.**

**To design the library, we first pooled all crRNA targeting nonessential control genes (141 crRNA targeting 50 genes). Then, for each paralog gene pair, we collected all crRNA pairs in both orientations – for n=3 crRNA per gene, there are n^2 = 9 crRNA pairs, or 18 total clones (A-B and B-A orientations for each). To generate single-knockout controls, we then took each crRNA targeting one of the paralogs and paired it with a crRNA randomly drawn from the nonessential pool, again designing clones in both A-B and B-A orientations, for a total of six control constructs per experimental gene (where n=3 crRNA/gene). Finally, we took our set of control essential genes (n=149 crRNA targeting 50 genes) and randomly paired each guide with a nonessential guide, in both orientations, as described above, for a total of 298 positive control guide constructs. The final library targets 841 genes (889 including nonessential genes) and 403 specified gene pairs with 12,328 constructs.**

*4. For estimating single KO effects, the authors include constructs in the library in which they pair the target gene guides with guides targeting known nonessential genes (line 155).*

*-- How were the non-essential genes selected?*

*-- How were the guides targeting the non-essential genes selected?*

*-- How many (target x neg) constructs were designed for each target gene?*

*-- Was the same set of non-essential guides used across target genes?*

See above for detailed library design criteria.

*5. A description of the methodology to go from sequencing reads to a matrix of counts is missing. Among others, the following information is missing:*

*-- What software was used to process sequencing reads? What were the parameters? What was the quality of the sequencing?*

*-- Which software (or programming language) was used to analyze the count data?*

*-- Was any normalization performed?*

*-- Were constructs with low counts in the plasmid library filtered out?*

We have added a detailed description of the analysis pipeline to the Methods section:

***Screen analysis***

**Construct sequences were combined into FASTA format (“paralog_2mer.fa”) and indexed with bowtie-build, and sequencing reads were mapped to this database with bowtie with the following command line parameters (trim ten 3’ bases, allow 3 mismatches, discard sequences which map to more than one reference sequence):**

**bowtie --trim3 10 -v 3 -m 1 -S --sam-nohead paralog_2mer [fastq_files] > [output.sam]**

**Sequence mapping rates ranged from 37-58%, averaging ~47%. Using this strict single-read mapping approach guarantees that only high-quality guide constructs were evaluated. Read counts were combined into a single matrix for further analysis (Supplementary Table 3).**

**Subsequent analysis was executed in Python notebooks, all of which are available at** [**https://figshare.com/articles/software/enCas12a_screen_analysis_pipeline/12275642**](https://figshare.com/articles/software/enCas12a_screen_analysis_pipeline/12275642)**. Mean read depth for all samples exceeded 500 reads/guide, and all samples showed read distributions with minimal skew (Supplementary Figure 1). A pseudocount of 5 reads was added to each construct in each sample, then read counts per sample were normalized to an average of 500 reads/guide (6.2M reads/sample), and log fold change for each guide was calculated relative to the plasmid sequence counts (notebook cas12a-step01-screen_QC). Screen replicate quality was verified by plotting the kernel density estimate of the fold changes of all control essential constructs vs. all other constructs (see notebook cas12a-step04_calc_SMF; summarized in Figure 3C). Screen-level fold change for each construct was then calculated as the mean of replicate fold changes.**

**Single mutant/knockout fitness, SMF, for each gene was calculated as the mean construct fold change of gene-control constructs, for both A and B positions. Construct-level consistency is shown in Figure 3D but gene level SMF is even more consistent (see notebook cas12a-step04_calc_SMF), with Pearson correlation coefficients ranging from 0.87 to 0.94. A-B and B-A constructs were subsequently averaged to calculate sample-level SMF for each gene. The distribution of each shows a left skew consistent with the dropout (negative SMF) of a proportion of the genes in the sample.**

**Difference in log fold change for a gene pair (dLFC) was calculated as observation, the mean LFC of all constructs targeting the gene pair, minus expectation, the sum of the SMF for the two genes. Given the skew of the SMF distributions, gene pairs with small positive SMF values sum yield an expectation of a positive LFC and, therefore, negative dLFC scores when the observed LFC is near zero. This explains the slight negative offset of dLFC distributions in Figure 3F and necessitates normalization before calling hits. We normalized by Z-transformation after removing the top and bottom 2.5% of scores (see notebook cas12a-step07_robustZ_of_dLFC). The resulting zdLFC table was used for all subsequent analysis of synthetic lethality.**

*6. In Figure 3F, the bulk of the distributions of the dLFC is shifted below 0 for all three cell lines. What is the reason behind this shift?*

See above description of screen analysis.

*7. How does the current work contrast to previously published CRISPR screens targeting pairs of paralogs? Previous work should be cited and discussed, for instance https://doi.org/10.1038/s41587-020-0437-z. The results of the present manuscript should be compared to he CRISPR-based screens from https://doi.org/10.1038/s41587-020-0437-z, at least for the pairs of genes that are common to both screens, and also contrasted to the dropout screen described in https://www.nature.com/articles/nbt.3834, which uses a comprehensive paired-gene library.*

We appreciate the reviewer’s comment and have attempted to address this point by performing the following analysis. We utilized the data provided for the paralog screen in <https://doi.org/10.1038/s41587-020-0437-z>. We note that 110 paralog pairs were common between two datasets.

For the 110 common paralog pairs, we performed comparisons between their HAP1 screen late time point to each of our screens.

Among the 24 synthetic lethal pairs we identified, 12 pairs were tested in the chymera screen. We compared our data from 3 screens with the HAP1 screen in the chymera paper.

We added these comparisons as a supplemental figure and the following section to our manuscript:

**The 678 paralog pairs evaluated in the CHyMErA screens contain 110 pairs targeted in our library, including 12 of the 24 hits we defined. Our results are generally consistent, with *TIA1/TIAL1, SAR1A/SAR1B, PITNA/PITNB*, and *CNOT7/CNOT8* scoring strongly in both assays (Supp Fig 4). In contrast, *MAPK1/MAPK3* and *CCNE1/CCNE2* are only hits in our cell lines.**

In the other manuscript mentioned by the reviewer (https://www.nature.com/articles/nbt.3834), we noticed that although a comprehensive paired-gene library was tested, only 3 pairs overlapped with our screen.

*8. The difference between the average dKO effects and the sum of sKO effects (dLFC) has been shown to be a suboptimal measure of synergy (https://doi.org/10.1186/s13059-019-1745-9) since it does not take into account guide-level variability. I strongly recommend the authors to use a statistical model that takes into account guide-level variability in the estimation of synergy scores, such as the GEMINI tool (https://doi.org/10.1186/s13059-019-1745-9), or using the methodology described in* [*https://www.nature.com/articles/nbt.3834*](https://www.nature.com/articles/nbt.3834)*.*

We applied the GEMINI tool to our data and show that for the Top 24 synthetic lethal hits we identified, the two scoring metrics show high correlation.

Across all data we observe similar correlations:

We have included these panels in a new Supplementary Figure 3 and added the following text to the Results section of the manuscript:

**Despite prior work suggesting simple difference in log fold change is not an effective measure of genetic interaction (30), we find that zdLFC is highly correlated with more detailed approaches such as GEMINI (30), with R^2^ values ranging from 0.59 (A549) to 0.74 (OVCAR8), and the two methods offer essentially no difference in hit calls (Supp Figure 3).**

*9. The Gaussian Mixture Modeling (Methods; 322-333) appears odd in that looking at Figure 1a, the "Sometimes" group (blue) does not look at all bivariate Gaussian, while the "Constitutive" group (orange) looks as if half its ellipsoid is empty. If this is used only to filter the list of paralog pairs, this may be relatively harmless (causing some false negatives in the discovery process, but not likely false positives) -- basically drawing a rectangle out to the right along the mean expression axis and low on the SD axis. Can the authors please comment on the intended purpose of the GMM within this work?*

We thank the reviewer for their feedback. We want to clarify that the use of the GMM within our work is to use modeling to define the set of constitutively expressed genes that are subsequently used to filter the list of paralog pairs, instead of using arbitrarily defined thresholds to define constitutive expression, (i.e.; mean expression>1, std dev of expression<1) to draw a rectangle. We believe that a model based approach reflects a more unbiased depiction of the actual data.

Moreover, we have added text in the methods section and a supplementary figure to show our reasoning with regard to using the 3-component GMM to identify subsets of genes in the data.

**Python 3.6.9 package sklearn and its GaussianMixture function was used to classify genes by Gaussian mixture modeling based on mean and standard deviation of mRNA expression. A three-component model was selected as the best fit to the data (Supp Fig 5) since the addition of a fourth component resulted in two highly overlapping component distributions.**

*10. Robust Z-score (~179-182): Details on precisely what this means? Is there normalization by a standard error, and if so, which one? I didn't see details in the Methods section. Was this based on winsorizing the dLFC values and centering and scaling them, or something else? We did not see these details.*

We simply used truncated data to calculate mean and standard deviation for a Z-transformation. We have added this text in the Methods section (notebooks are available on Figshare):

**We normalized by Z-transformation after removing the top and bottom 2.5% of scores (see notebook cas12a-step07_robustZ_of_dLFC). The resulting zdLFC table was used for all subsequent analysis of synthetic lethality.**

*11. Line 232: Is the factor "3" from comparing "27.9%" to "11.6%"? If so, suggest "2.4" instead of "3". If not, where does "3" come from?*

We appreciate the reviewer’s suggestion and their question. This factor comes from an earlier analysis we had performed. Among constitutively expressed genes, we evaluated the fraction of never essentials with paralogs to that of common essentials with paralogs based on paralog sequence similarity. We observed that at 45% sequence similarity or higher, never essentials (18.3%) were 3 times more likely as common essentials (6.1%) to have a paralog. However we did not address this in the manuscript, and we have amended the statement in the Conclusions to say “They are more than twice as likely…”

*12. Methods (341-346): "... the percent sequence identities of all human target genes to their paralogs were plotted against the percent sequence identities of the paralogs to the target human gene to reveal that the majority of the human paralogous gene pairs had low percentage sequence similarity." It sounds as if they are getting the percent similarity of "B" to "A", then of "A" to "B", then plotting them against each other? If so, this sounds good, but this description is a little confusing -- consider rewording?*

We thank the reviewer for their feedback and agree that our explanation is confusing. To address this, we changed the description as follows:

**After removing duplicate gene pairs and filtering for constitutively expressed genes, for each paralog pair (A-B pair), we obtained their percent sequence identities and we plotted the sequence similarities of A to B against those of B to A. We observed that the majority of the human paralogous gene pairs had low percentage sequence similarity.**

*13. Possible typos or awkward constructions:*

*-- Line 150: "We manually five additional candidate genes…"*

*-- LIne 184: "Show characteristics lethal synthality…"*

*-- Line 345: "... paralog pairs which were both in always expressed gene list"*

*-- Line 359-360: "...synthetic essentiality which is defined that a gene is essential when the other paralog partner lost the function…."*

We thank the reviewer for noticing the typos in our manuscript. We made the following changes:

-- Line 164-165: "We manually added five additional candidate genes…"

-- LIne 206-207: "Show characteristics of synthetic lethality…"

-- Line 353-354: "... paralog pairs which were both constitutively expressed"

-- Line 369-370: "...synthetic essentiality which is defined when a gene is observed as essential when its paralog partner loses its function…."

**Reviewer #2: See joint review with Reviewer 1**

***Reviewer #3:*** *In this manuscript the authors explore the role of functional buffering provided by the paralogs of a gene in compensating its activity when that gene is inactivated via CRISPR-cas9 targeting. Thus, shedding light on how this prevents constitutively expressed genes to be detected as 'essential' for cellular survival in large scale mono-genic knock down screens.*

*The authors build on the assumption that constitutively expressed genes are essential in at least some context and that combinations of environmental or genetic buffering masks the impact of their experimental inactivation on cellular fitness. By mining public available cancer dependency datasets, encompassing gene essentiality profiles of hundreds of cell lines, the authors found that paralog gene pairs are over represented among constitutively expressed genes that are never called as significantly essential, i.e. not impacting cellular fitness upon CRISPR-cas9 targeting. This supports the authors hypothesis hinting that the genetic buffering compensating the inactivation of constitutively expressed genes is provided by their paralogs.*

*The authors then reasoned that simultaneously probing cellular models for their dependency on paralog pairs might offer an opportunity to identify new synthetic lethal gene pairs that might be exploited for the development of new cancer therapies.*

*To this aim, the authors have selected 400 hundreds candidate paralog gene pairs, according to different criteria and performed a dual knock out screen targeting each pair in 3 immortalised human cancer cell lines through a enCas12a dual knockout system. 24 of the tested pairs resulted into a synthetic lethality in at least 2 of the tested models and are described in dept by the authors.*

*Briefly, this manuscript describes important findings that might have an impact on how outcomes from monogenic knock out screens will be interpreted and illustrates a strategy for identifying promising synthetic lethality to be further confirmed via dedicated dual knock out screens. Genetic interaction screens are expected to be increasingly performed in the future in human cells, so providing guidance on which are the gene pairs to be prioritised for testing across a so large spectrum it is timely and important.*

*However, the hypothesis tested by the authors has been already investigated, using a similar approach, in [PMID: 31652272]. In addition the selection of 400 gene pairs tested with the dual knock out screen performed by the authors is not guided by the CRISPR based landmark analysis nor the buffering association study. As a consequence, it seems to me that the analyses reported in the first part of the results and figures 1 and 2 are just confirmatory and could be totally removed.*

We appreciate the reviewer’s critical eye, but we strongly disagree with this assertion. Our manuscript describes both computational and experimental approaches to identifying paralog buffering. First, both these approaches rely on establishing the hypothesis that paralog buffering exists. We are eager proponents of the work published by De Kegel and Ryan and agree that their observations lay a strong foundation for our work. However, simply because a topic has been broached once in the literature does not prevent it being subsequently confirmed - in fact, just the opposite. Confirmatory results are critical to the scientific enterprise as a whole; indeed it is the lack of confirmatory results that are most damning of some of the most high-profile errors in life sciences.

Second, our computational approach to identifying paralog synthetic lethals relies on mutation-driven loss of function among constitutively expressed genes and is therefore orthogonal to the approach of De Kegel & Ryan. The differences in our results highlight the importance of applying different methodologies to the same problem. We have added this explicit comparison to the main text:

**Unfortunately, the cell lines screened by CRISPR knockout libraries only contain LOF alleles of a fraction of the candidate paralogs, limiting this discovery avenue to a few dozen pairs. A comparison with De Kegel and Ryan shows that more than half of our computationally derived hits (39 of 66, 59%; Supplementary Figure) are present in their study, indicating strong concordance between the two approaches. Nevertheless, the large number of hits unique to each approach clearly indicates that neither approach is saturating, and additional approaches, both computational and experimental, are required to build our catalog of paralog synthetic lethals.**

*-In addition, the title states that Multiplex enCas12a screens show… etc etc. whereas this is demonstrated by the authors via mining existing data. As a consequence the title should be changed.*

We agree that the original title is very confusing. We have changed it to “**Multiplex enCas12a screens detect functional buffering among paralogs otherwise masked in monogenic Cas9 knockout screens”.**

*The results from the dual knock out screen remains potentially very useful and potentially impactful but further functional characterisation of the identified synthetic lethal gene pairs should have been performed.*

In the Covid-19 age, we find it exceptionally difficult to perform robust lab work. We note that these screens were completed before the widespread lab shutdowns. We have made every effort to include computational validation of our experimental results, and in addition we have included comparisons with another recently published survey of genetic interactions among paralogs.

*In conclusion, I do not believe that this manuscript would be suitable for publication in Genome Biology at this stage. The author should clarify scope and aim of this manuscript. I would definitely reshape this submission and focus it on the dual screen effort, whose outcomes deserve definitely a closer look.*

*In addition, I have the following major points:*

** From the main text it seems that the author detected unbiasedly three major populations of genes based on their mean basal expression (and corresponding variability) across cell lines. By looking at figure 1A and reading the methods, it is clear that the number of Gaussian distributions to be used was decided a priori by the authors. This should be made clear in the main text or a numerical justification for this choice should be provided. In addition, from figure 1A it seems that several points are drawn from the 'orange distribution' even if they have a mean expression level proximal to 0 and a quite low standard deviation. Can these genes be really considered constitutively expressed?*

We thank the reviewer for their comment. The use of the GMM within our work is to use a model-based approach to define the set of constitutively expressed genes which are the genes expressed across a lot of tissues with low variation. These constitutively expressed genes were then used to filter the list of paralog pairs so that pairs that show tissue specific expression would be excluded from the analysis. As the reviewer mentioned, the number of Gaussian distributions used was chosen as 3 since fits with two or four components did not, in our view, accurately model the data. We have included an explanation and a supplementary figure describing our justification (see response to reviewer 1 / 2 above).

With regard to the outliers in the “orange distribution” - such is the nature of modeling. We judge this to be a more robust and less biased approach than simply drawing lines on a map, but the tradeoff is that the models are perhaps not as accurate at the edges as we would hope. We are confident that, had we chosen an arbitrary mean vs. std dev cutoff, a reviewer would have declared this unacceptable and insisted on a model-based approach!

** The author call the 'sometimes expressed genes' tissue-specific. I don't think that this is justified in any way. Some of these genes could be just expressed in minority of cell lines across different tissues, or ubiquitous subtypes with a common mutated oncogene, thus same set of active transcription factors*

We agree with the reviewer’s observation and we have changed the text: “genes that show variable, sometimes tissue-specific gene expression (“sometimes expressed” genes, high variance)”.

** no statistical estimation is provided with respect to paralog enrichments across the different classes of genes in figure 1B, other than percentages. This should be included.*

We make no statement as to the enrichment for paralogs in the bins in Figure 1B, and therefore no statistical test is warranted (or appropriate). We do, however, imply that never-essentials are enriched for paralogs relative to always-essentials in Figure 1D, without statistical basis. We have corrected this by calculating a P-value based on the Z-test for difference in proportions at the 35% sequence identity we specify in the figure (P<10^-89^), and we have added this statement in the main text.

** FDRs and effect size indicators for the tested buffering interactions reported in figure 2 should be provided*

We thank the reviewer for their feedback. We have added FDR and odds ratio of buffering interactions in Figure 2 to Supplementary Table 1.

** The author should discuss the possibility of the identified synthetic lethal gene pairs being context-specific, and how the genomic heterogeneity of the cell line selected for their study might have impacted the results. Furthermore, which criteria were used for selecting those three cell lines?*

As noted in the text, the cell lines were chosen in part because of their different backgrounds (lung, colorectal, ovarian cancer). Naturally we expect that some fraction of our synthetic lethals would be context dependent; in fact this is one of the messages conveyed in Figure 4. A full paragraph in the Results section details this thinking:

**Importantly, we note that synthetic lethality, even among paralogs, can also be context-dependent. Cyclin paralogs are often redundant interaction partners with their cognate cyclin-dependent kinases; here, CCNE1 and CCNE2 are synthetic lethal where CDK2 is highly essential, especially in OVCAR8 (Figure 4F). Similarly, CCNT1-CCNT2 show weaker but significant synthetic lethality (zdLFC < -2.5 in A549 and < -1 in the other two cell lines) while their binding partner, CDK9, is highly essential in all three (Figure 4G). Though the synthetic lethal relationships between SWI/SNF complex members *ARID1A/ARID1B* and *SMARCA2/SMARCA4* are well described in the literature and are detected in large scale screening data, their synthetic lethality only occurs where the SWI/SNF complex is itself essential. We test four paralog pairs in the BAF complex: *ARID1A/ARID1B, SMARCA2/SMARCA4, SMARCC1/SMARCC2,* and *SMARCD1/SMARCD2*, but we detect no synthetic lethal interactions, most likely because the complex itself is not essential in the cell lines we tested.**

** The Broad DepMap dataset is slightly outdated. A 20Q2 version is now available. The authors might consider revamping their analysis with this more recent version or (even better) by using a recent joint dataset of cancer dependencies integrating both Sanger and Broad CRISPR screens (available at* [*https://depmap.org/broad-sanger/*](https://depmap.org/broad-sanger/)

We appreciate the reviewer’s comment. To address the reviewer’s concerns, we repeated our analysis by incorporating all CCLE cell line expression data from 1303 cell lines to define constitutively expressed genes with the 20Q2 essentiality data. However we observed that this analysis did not change the trends we described in the manuscript.

** I have definitely a problem with the title of this manuscript as it also suggests to me the opposite of the finding presented by the authors. Doesn't 'functional buffering systematically absent in genome-wide CRISPR/Cas9 screens' imply that the effect of the buffering is absent therefore in such screens paralogs can be detected as essential genes? What about to replace this with something more like: "Functional buffering prevents detecting fitness genes among paralogs in genome-wide CRISPR/Cas9 etc. etc."?*

As noted above, we have changed the title.

*Minor points:*

** The color scheme in figure 1B could be improved, for example I cannot decide if the top bar in the 'sometimes expressed genes' column corresponds to common or context essential.*

We have added cross-hatching to clarify the differences in the gene classes.

**Second round of review**

**Reviewer 1**

I reviewed the author's responses to the several concerns we raised in the initial submission and appreciate the work they have done to address them.  I do not have further substantive concerns.

**Reviewer 2**

We congratulate the reviewers for the substantial work that they have put into their reviewed manuscript.

**Reviewer 3**

The authors have convincingly responded to my concerns, further motivating their in-silico analysis of paralogue buffering effects, comparing their results with those presented in a previous study showing concordance but also results' complementarity/expansion, and modified the main text according to my observations and requests of further clarification / specification of statistical outcomes.
